# Resuscitation room management of patients with non-traumatic critical illness in the emergency department (OBSERvE-DUS-study)

**DOI:** 10.1186/s12873-023-00812-y

**Published:** 2023-04-17

**Authors:** Janina Dziegielewski, Falko C. Schulte, Christian Jung, Georg Wolff, Oliver Hannappel, Philipp Kümpers, Michael Bernhard, Mark Michael

**Affiliations:** 1 Emergency Department, Medical Faculty, Heinrich Heine University, Moorenstrasse 5, 40225 Duesseldorf, Germany; 2 Division of Cardiology, Pulmonology and Vascular Medicine, Medical Faculty, Heinrich Heine University, Moorenstrasse 5, 40225 Duesseldorf, Germany; 3 Information, Communication and Medicine Technology, University Hospital Duesseldorf, Moorenstrasse 5, 40225 Duesseldorf, Duesseldorf, Germany; 4 Department of Medicine D, Division of General Internal and Emergency Medicine, Hypertension and Rheumatology, University Hospital Münster, Albert-Schweitzer-Campus 1, 48149 Nephrology, Münster, Germany

**Keywords:** Epidemiology, Critical interventions, Critically ill non-traumatic patients, Emergency department

## Abstract

**Background:**

Few studies address the care of critically ill non-traumatic patients in the emergency department (ED). The aim of this study was to assess the epidemiology, management, and outcome of these patients.

**Methods:**

In this retrospective study, we identified and analyzed data from all consecutive adult critically ill non-traumatic ED patients treated from March 2018 to February 2019. Patient characteristics, major complaint leading to admission, out-of-hospital, and in-hospital interventions and 30-day mortality were extracted from medical records of the electronic patient data management system.

**Results:**

During the study period, we analyzed 40,764 patients admitted to the ED. Of these, 621 (1.5%) critically ill non-traumatic patients were admitted for life-threatening emergencies to the resuscitation room (age: 70 ± 16 years, 52% male). Leading problem on admission was disability/unconsciousness (D), shock (C), respiratory failure (B), airway obstruction (A), and environment problems (E) in 41%, 31%, 25%, 2%, and 1%, respectively. Out-of-hospital and in-hospital measures included: intravenous access (61% vs. 99%), 12-lead ECG (55% vs. 87%), invasive airway management (21% vs. 34%) invasive ventilation (21% vs. 34%), catecholamines (9% vs. 30%), arterial access (0% vs. 52%), and cardiopulmonary resuscitation (11% vs. 6%). The underlying diagnoses were mainly neurological (29%), followed by cardiological (28%), and pulmonological (20%) emergencies. The mean length of stay (LOS) in the resuscitation room and ED was 123 ± 122 and 415 ± 479 min, respectively. The 30-day mortality was 18.5%.

**Conclusion:**

The data describe the care of critically ill non-traumatic patients in the resuscitation room. Based on these data, algorithms for the structured care of critically ill non-traumatic patients need to be developed.

**Supplementary Information:**

The online version contains supplementary material available at 10.1186/s12873-023-00812-y.

## Background

The incidence of emergency medical services (EMS) rescue missions is continuously increasing in Germany nationwide [1]: A complete survey of the numbers of EMS for several states and extrapolations of those numbers for all of Germany from 2009 to 2018 show annual growth rates of about 5%.

Both traumatic and non-traumatic emergencies are treated in out-of-hospital setting, with the emergency department (ED) providing the interdisciplinary and integrative interface between out-of-hospital and early inpatient care for patients with life-threatening conditions of any cause [2, 3]. In Germany, EMS patients as well as walking emergencies with life-threatening conditions are preferentially admitted to the acute area of an ED (the so-called “shock room” or “resuscitation room”).

For trauma patients, there is a transsectoral and structured care in Germany with clear guidelines for admission in the ED resuscitation rooms and the corresponding in-hospital follow-up care. The trauma registry of the German Society for Trauma Surgery (DGU, www.traumaregister.de), which has been established since the year 1997, provides a comprehensive evaluation of the care of severely injured patients.

Care concepts for critically ill non-traumatic patients at the interface between out-of-hospital care, ED, and in-hospital care (mainly on intensive care units) are only available for a few specific conditions (e.g., ST elevation myocardial infarction, sepsis, stroke), but not for the broad mass of unselected critically ill non-traumatic patients. Therefore, epidemiological data for the transsectoral care of critically ill non-traumatic patients are scarce [2].

Unfortunately, there are few studies in Germany that have addressed the epidemiology and care of critically ill non-traumatic patients in the ED [4–7). Due to insufficient data on this vulnerable patient population, there is a lack of attention and recommendations for the management of critically ill non-traumatic patients in the ED.

The aim of this study was to collect additional data on the epidemiology, management, and outcome of critically ill non-traumatic patients in another large university ED and to compare these with existing studies from two other German sites.

## Methods

### Study design

In this retrospective single-center cohort study [*Observation of critically ill patients in the resuscitation room of the Emergency Department in Duesseldorf* (OBSERvE-DUS)-study] all consecutive critically ill non-traumatic patients admitted to the resuscitation room of the ED of the tertiary-care University Hospital Duesseldorf, Germany from 01 March 2018 to 28 February 2019 were identified and analyzed. The study protocol was approved by the Ethics Committee of the Medical Faculty of the Heinrich Heine University of Duesseldorf, Germany (No. 2020 − 960).

### Setting

The catchment area of our university hospital covers the city area (217 km^2^) with a population of around 650,000. In addition, the university hospital is the main provider hospital for the neighboring rescue service areas for certain issues. The out-of-hospital emergency medicine service in Düsseldorf, Germany, is provided from eight locations and 15 emergency vehicles. In the reference period of the year 2019, the average age in the state capital of Düsseldorf, Germany, was 43 years. The structural change within the group of the older people can be seen in the Greying Index. It shows the ratio of 80-year-olds and older to 65- to 79-year-olds and shows an upward trend [8].

More than 40,000 patients are managed annually in the ED, of which about 60% have non-traumatic acute diseases or emergencies. Due to local standard operating procedures, only few of the patients bypass the ED for specific interventions (e.g., thrombectomy treatment for acute stroke, percutaneous coronary intervention for ST-segment elevation myocardial infarction).

Four specially equipped resuscitation rooms are available for critically ill non-traumatic and trauma patients. Here, intensive medical measures (e.g., airway management, mechanical ventilation, cardiovascular therapy, invasive circulation monitoring) can be carried out immediately. The measures are simultaneously documented in the patient data management system (PDMS). In addition, there are twelve cabins equipped with oxygen connections, medical monitors, nursing trolleys and computer workstation according to the latest standards. A holding area with six monitored beds and an additional emergency admission unit with twelve beds are integrated into the ED. Numerous emergency trolleys with additional medical and technical equipment are available for immediate treatment of acute emergencies in the ED.

Out-of-hospital care is provided by a two-tier EMS system staffed with paramedics and EMS physicians. In the ED, critically ill non-traumatic patients are treated by a team of two nurses, one resident and one attending physician with emergency and intensive care expertise. Other specialists are consulted as necessary.

### Study definitions and data collection

All adult critically ill non-traumatic patients ≥ 18 years of age admitted to the ED resuscitation room were included. Epidemiological and medical care data were anonymously aggregated from the PDMS (COPRA®, COPRA System GmbH, Berlin, Germany) and the hospital information system (MEDICO®, Cerner Deutschland GmbH, Itstein, Germany) by database query and transferred to a spreadsheet program (Microsoft® Office 365, version 16.37, Microsoft Corporation, Redmond, USA). It was no longer possible to draw conclusions about the individual case with this data. Thus, the requirements for data protection according to the German Data Protection Regulation (DSGVO) were fulfilled and the guidelines of Good Clinical Practice (GCP) were adhered to.

Patients were included in this study through a stepwise identification procedure with (1) treatment in one of the four available resuscitation room, (2) fulfillment at least one criteria of the resuscitation room admission list (Table S1) and (3) manual screening of the medical data.

Responsibility for documentation rest with the ED physicians and nurses. During the ED stay, continuously and paperless electronically documentation was performed, as well as vital sign recording in the electronic database.

The ED resuscitation room evaluation chart includes the patients’ characteristics (e.g., age, sex, weight, size). The triage classification and the vital signs at ED admission and resuscitation room discharge [e.g., systolic blood pressure (mmHg), heart rate (beats per minute (bpm)), and peripheral oxygen saturation (SpO2 in %)] were recorded. As predefined target ranges of vital signs as surrogate parameters were 100–150 mmHg for systolic blood pressure (SBP), heart rate (HR) between 50 and 100 bpm, and oxygen saturation (SpO2) above 94%.

Out-of-hospital EMS management includes prior notification before ED admission using the ABCDE approach. Prior airway management, ventilation, and administration of catecholamines are also included here. The EMS protocol includes the qualifications of the personnel (paramedic ± out-of-hospital emergency physician) admitting patients to the ED. The risk score based on the National Advisory Committee of Aeronautics (NACA) score, the preliminary diagnosis, the leading ABCDE problem, and any medical interventions were taken from the EMS protocol. The American Society of Anesthesiologists (ASA) score was determined retrospectively.

The out-of-hospital cardiac arrest (OHCA) reporting follows the Utstein definition as closely as possible [9].

### In-hospital management, time steps and outcomes

During management in the resuscitation room, definitive time points of events and interventions were recorded in the PDMS (e.g., time of admission, end of handover, time of first blood pressure measurement). The outcomes studied were length of stay (LOS) in the resuscitation room, LOS in the ED, allocation to the intensive care unit and all-cause mortality at day 30.

### Comparison with previous published studies

To provide a good overview to the already published studies, we make a comparison between the current OBSERvE-DUS study and three other studies from other sites [4, 6, 7]. The preceding OBSERvE-1 [6] and OBSERvE-2 [7] studies care prospectively conducted data collections from 2014–2015 and 2017–2018 at the ED of the university hospital of Leipzig, Germany. The data of 532 and 457 critically ill non-traumatic patients were documented using a standardized OBSERvE-item collection, respectively. Documentation took place simultaneously with acute resuscitation room care carried out by a senior ED physician with expertise in emergency and critical care medicine. Another single-center retrospective cohort study [4] collected data from 193 critically ill non-traumatic patients in 2018–2019 in the ED of a teaching hospital in Mönchengladbach, Germany, the team leader filled out a standardized paper-based questionnaire mainly included the standardized OBSERvE-item collection, and qualified the patients as a resuscitation room patient.”

### Statistical analysis

Data are presented as numbers and percentages, mean ± standard deviation (SD), median with minimal and maximal values as appropriate. The chi square-test, the student’s t-test, and the Wilcoxon rank-sum test were used to compare groups as appropriate. All tests used were twosided, and statistical significance was set at *p* < 0.05. Microsoft® Office 365 (version 16.37, Microsoft Corporation, Redmond, USA) and DataGraph 5.0 (Visual Data Tools, Inc. 2006–2022) were used for statistical analyses and to prepare figures.

## Results

During the study period, a total of 40,764 patients were treated in the ED, of whom 23,235 patients (57.0%) were admitted for non-traumatic emergencies. Out of all ED patients, 5,206 patients were treated in one of the four available resuscitation rooms, and 1,233 patients met at least one criteria of the resuscitation room admission list from which medical records were manually screened. Of these, 630 critically ill non-traumatic patients (1.5% of all patients in the study period) received medical treatment in the ED resuscitation room because of significant ABCDE problems. Nine patients were excluded due to incomplete records due to IT issues caused delayed documentation of each procedure and times could not be accurately tracked, procedures were not documented, or late data entry occurred with inconsistent times. Data from 621 patients (98.6% of all critically ill non-traumatic patients in the study period) were available for the final analysis.

### Patient’s characteristics

The main findings on patient characteristics, including the leading ABCDE problem, are shown in Table 1.

**Table 1:** Comparison between the current OBSERvE-DUS study and three studies from other German sides: Study site information, patient´s characteristics, and vital signs in studies concerning resuscitation room management of patients suffer from non-traumatic critical illness

	OBSERvE-DUS n = 621	OBSERvE-1 [6] n = 532	OBSERvE 2 [7] n = 457	Kreß et al. [4] n = 193	p (*Student-t-test, #χ²-test)
**Study information**					
Study design	single-center retrospective cohort study	single-center prospective observational cohort study	single-center prospective observational cohort study	single-center retrospective cohort study	
Study period (months)	12	12	12	6	
Study setting (type)	tertiary-care university hospital	tertiary-care university hospital	tertiary-care university hospital	academic teaching hospital	
Case load (n)	40,764	34,303	35,039	19,854	
Case load of critically ill non-traumatic patients [n, (%)]	621 (1.5)	532 (1.6)	457 (1.3)	193 (1.0)	^#a^0.8909, ^#b^0.7836, ^#c^0.6034
**Patient´s characteristics**					
Age (years, MV ± SD)	70 ± 16	67 ± 17	65 ± 17	66 ± 16	^*a^0.0021, ^*b^<0.0001, ^*c^0.0025
Sex, male [n, (%)]	323 (52.0)	310 (58.3)	273 (59.7)	106 (54.9)	^#a^0.1114, ^#b^0.0597, ^#c^0.6042
NACA [n, (%)]	539 (86.8)	489 (92.0)	388 (85.0)	not reported	^#a^0.0072, ^#b^0.4355, ^#c^ n.a.
Admission by EMS [n, (%)]	578 (93.1)	498 (93.6)	438 (93.7)	not reported	^#a^0.7432, ^#b^0.7038, ^#c^ n.a.
**ABCDE problems [n, (%)]**					
A (airway)	12 (1.9)	20 (3.8)	17 (3.7)	not reported	^#a^0.7672, ^#b^0.7818, ^#c^n.a.
B (breathing)	152 (24.5)	141 (26.5)	132 (28.8)	not reported	^#a^0.6951, ^#b^0.4803, ^#c^n.a.
C (circulation)	194 (31.2)	189 (35.5)	160 (35.1)	not reported	^#a^0.3727, ^#b^0.4379, ^#c^n.a.
D (disability)	256 (41.2)	177 (33.3)	146 (31.9)	not reported	^#a^0.0963, ^#b^0.0648, ^#c^n.a.
E (environment)	7 (1.1)	5 (0.9)	2 (0.4)	not reported	^#a^0.9740, ^#b^0.9322, ^#c^n.a.
**Vital signs admission [MV ± SD]**					
Systolic blood pressure (mmHg)	129 ± 45	135 ± 44	136 ± 40	131 ± 45	^*a^0.0228, ^*b^0.0083, ^*c^0.5898
Heart rate (bpm)	95 ± 35	96 ± 30	99 ± 32	95 ± 33	^*a^0.6058, ^*b^0.0548, ^*c^1.0000
Shock index (bpm/mmHg)	0.8 ± 0.5	0.8 ± 0.5	0.8 ± 0.4	not reported	^*a^1.0000, ^*b^1.0000,^*c^n.a
Oxygen saturation (%)	94 ± 8	92 ± 11	94 ± 10	91 ± 10	^*a^0.0004, ^*b^1.0000, ^*c^<0.0001
Respiratory rate (min^− 1^)	22 ± 13	20 ± 10	20 ± 9	22 ± 9	^*a^0.0039, ^*b^0.0048, ^*c^1.0000
Glasgow coma score (points)	10 ± 5	8 ± 5	8 ± 5	11 ± 4	^*a^<0.0001, ^*b^<0.0001,^*c^0.0114
Temperature tympanal (°C)	36 ± 2	36 ± 1	37 ± 2	not reported	^**a^1.0000, ^*b^<0.0001, ^*c^n.a
ROSC at admission [n, (%)]	52 (71.2)	55 (60.4)	43 (47.3)	not reported	^#a^<0.0001, ^#b^<0.0001, ^#c^n.a

NACA = National Advisory Committee of Aeronautics; EMS = Emergency Medical Services; MV = mean value; SD standard deviation, C = Celsius; CPR = cardiopulmonary resuscitation, ^a^comparison between OBSERvE-DUS and OBSEvE-1, ^b^comparison between OBSERvE-DUS and OBSEvE-2, comparison between OBSERvE-DUS and Kreß et al., n.a. = not available

The mean age of the patients was 70 ± 16 years. Women and men were equally distributed (47.0 vs. 52.0%, p = 0.139). Men were significantly younger than women [69 ± 15 vs. 72 ± 17 years, p = 0.02]. The proportion of women aged 80 years and older was significantly higher than that of men (37.9 vs. 28.5%, p = 0.01). Patient were admitted outside (16:00 and 07:59 h) and within (08:00 and 15:59 h) the core working hours in 52 vs. 48%, respectively. During the week (70%) and at weekends (30%) an average of 87 ± 15 and 94 ± 11 critically ill non-traumatic patients were admitted to the ED, respectively. Most patients were admitted to the resuscitation room by EMS (93.1%), followed by ED triage of walking emergencies (3.5%), interhospital transport (2.7%), and in-hospital medical emergency teams (0.6%).

### Out-of-hospital and in-hospital emergency medical care

The frequency of different aspects of emergency care provided by EMS and the ED team in the resuscitation room is shown in Table 2. While in the ED resuscitation room, several diagnostic procedures were performed to determine the underlying problem.

**Table 2:** Emergency life-saving interventions and diagnostic procedures in studies concerning resuscitation room management of patients suffer from non-traumatic critical illness. Comparison between the current OBSERvE-DUS study and three studies from other German sides

Emergency life-saving interventions and diagnostic procedures	OBSERvE-DUS n = 621		OBSERvE-1 [6] n = 532	OBSERvE 2 [7] n = 457	Kreß et al. [4] n = 193	p (χ²-test)
**Out-of-hospital EMS setting [n, (%)]**						
venous access	379 (61.0)		515 (96.8)	404 (88.4)	not reported	^a^<0.0001, ^b^<0.0001, ^c^n.a.
12-lead-ECG	344 (55.4)		266 (50.0)	200 (43.8)	not reported	^a^0.0672, ^b^0.0002, ^c^n.a.
airway management	125 (20.1)		163 (30.6)	158 (34.5)	not reported	^a^<0.0001, ^b^<0.0001, ^c^n.a.
mechanical ventilation	125 (20.1)		160 (30.1)	158 (34.5)	not reported	^a^0.0001, ^b^<0.0001, ^c^n.a.
catecholamines	56 (9.0)		128 (24.0)	88 (19.3)	not reported	^a^<0.0001, ^b^<0.0001, ^c^n.a.
CPR	73 (11.8)		98 (18.4)	92 (20.1)	not reported	^a^0.0017, ^b^0.0002, ^c^n.a.
non-invasive ventilation	35 (5.6)		37 (7.0)	57 (12.5)	not reported	0.3276, ^b^<0.0001, ^c^n.a.
intraosseous access	21 (3.4)		18 (3.4)	12 (2.6)	not reported	^a^1.0000, ^b^0.4513, ^c^n.a.
arterial line	0 (0.0)		1 (0.2)	0 (0.0)	not reported	^a^1.0000, ^b^1.0000, ^c^n.a.
rescue thrombolysis	1 (0.2)		3 (0.6)	7 (1.5)	not reported	^a^0.2742, ^b^0.0146, ^c^n.a.
chest tube	0 (0.0)		1 (0.2)	0 (0.0)	not reported	^a^1.000, ^b^1.000, ^c^n.a.
therapeutic hypothermia	0 (0.0)		4 (0.8)	0 (0.0)	not reported	^a^1.000, ^b^1.000, ^c^n.a.
ACCD	8 (1.3)		18 (3.4)	41 (9.0)	not reported	^a^0.0170, ^b^<0.0001, ^c^n.a.
**ED resuscitation room setting [n, (%)]**						
venous access	617 (99.4)		409 (76.9)	352 (77.0)	not reported	^a^<0.0001, ^b^<0.0001, ^c^n.a.
12-lead-ECG	540 (87.0)		460 (86.5)	362 (79.2)	not reported	^a^0.8028, ^b^0.0006, ^c^n.a.
airway management	212 (34.1)		141 (27.1)	107 (23.4)	56 (29.0)	^a^0.0104, ^b^0.0001, ^c^0.1880
mechanical ventilation	211 (34.0)		304 (57.2)	251 (55.0)	56 (29.0)	^a^<0.0001, ^b^<0.0001, ^c^0.1966
catecholamines	189 (30.4)		128 (24.1)	144 (31.5)	48 (24.9)	^a^0.0170, ^b^0.6994, ^c^0.1420
CPR	40 (6.4)		65 (12.2)	60 (13.1)	19 (9.8)	^a^0.0006, ^b^0.0002, ^c^0.1108
non-invasive ventilation/HFNC	73 (11.7)		87 (16.4)	89 (19.5)	60 (31.1)	^a^0.0214, ^b^0.0004, ^c^<0.0001
intraosseous access	4 (0.6)		1 (0.2)	3 (0.7)	6 (3.1)	^a^0.2927, ^b^0.8391, ^c^0.0052
arterial line	320 (51.5)		309 (58.1)	245 (53.6)	78 (40.4)	^a^0.0249, ^b^0.4953, ^c^0.0071
central venous line	193 (31.1)		not reported	not reported	8 (4.2)	^a^n.a., ^b^n.a.^c^<0.0001
rescue thrombolysis	80 (12.9)		13 (2.4)	5 (1.1)	not reported	^a^<0.0001, ^b^<0.0001, ^c^n.a.
chest tube	23 (3.7)		0 (0.0)	4 (0.9)	4 (2.1)	^a^n.a., ^b^0.0037, ^c^0.2789
therapeutic hypothermia	2 (0.3)		20 (3.8)	5 (1.1)	not reported	^a^<0.0001, ^b^0.1035, ^c^n.a.
ACCD	11 (1.8)		19 (3.6)	43 (9.4)	not reported	^a^0.0570, ^b^<0.0001, ^c^n.a.
**ED Diagnostic procedures [n, (%)]**						
blood samples	609 (98.1)		496 (93.2)	383 (83.8)	not reported	^a^<0.0001, ^b^<0.0001, ^c^n.a.
blood gas analysis	586 (94.4)		496 (93.2)	383 (83.8)	not reported	^a^0.3982 ^b^<0.0001, ^c^n.a.
blood cultures	217 (34.9)		45 (8.5)	60 (13.1)	not reported	^a^<0.0001, ^b^<0.0001, ^c^n.a.
abdominal sonography (FAST)	102 (16.4)		not reported	not reported	not reported	^a,b,c^n.a.
echocardiography	291 (46.9)		149 (28.0)	166 (36.3)	26 (13.5) ^c^	^a^<0.0001, ^b^0.0007, ^c^<0.0001
chest x-ray	313 (50.4)		227 (42.7)	126 (27.6)	not reported	^a^0.0090, ^b^<0.0001, ^c^n.a.
CT/MRI	331 (56.8)		227 (42.7)	167 (36.5)	65 (33.7) ^c^	^a^<0.0001, ^b^<0.0001, ^c^<0.0001
gastroscopy	5 (0.8)		not reported	not reported	not reported	^a,b,c^n.a.

EMS = emergency medical services; ECG = electrocardiogram; CPR = cardiopulmonary resuscitation; HFNC = high flow nasal cannula; ACCD = automated chest compression device; FAST = focused assessment with sonography in trauma; CT = computered tomography; MRI = magnetic resonance imaging, ^a^comparison between OBSERvE-DUS and OBSEvE-1, ^b^comparison between OBSERvE-DUS and OBSEvE-2, ^c^comparison between OBSERvE-DUS and Kreß et al., n.a. = not available

### Vital signs at hospital admission

**Fig. 1:**
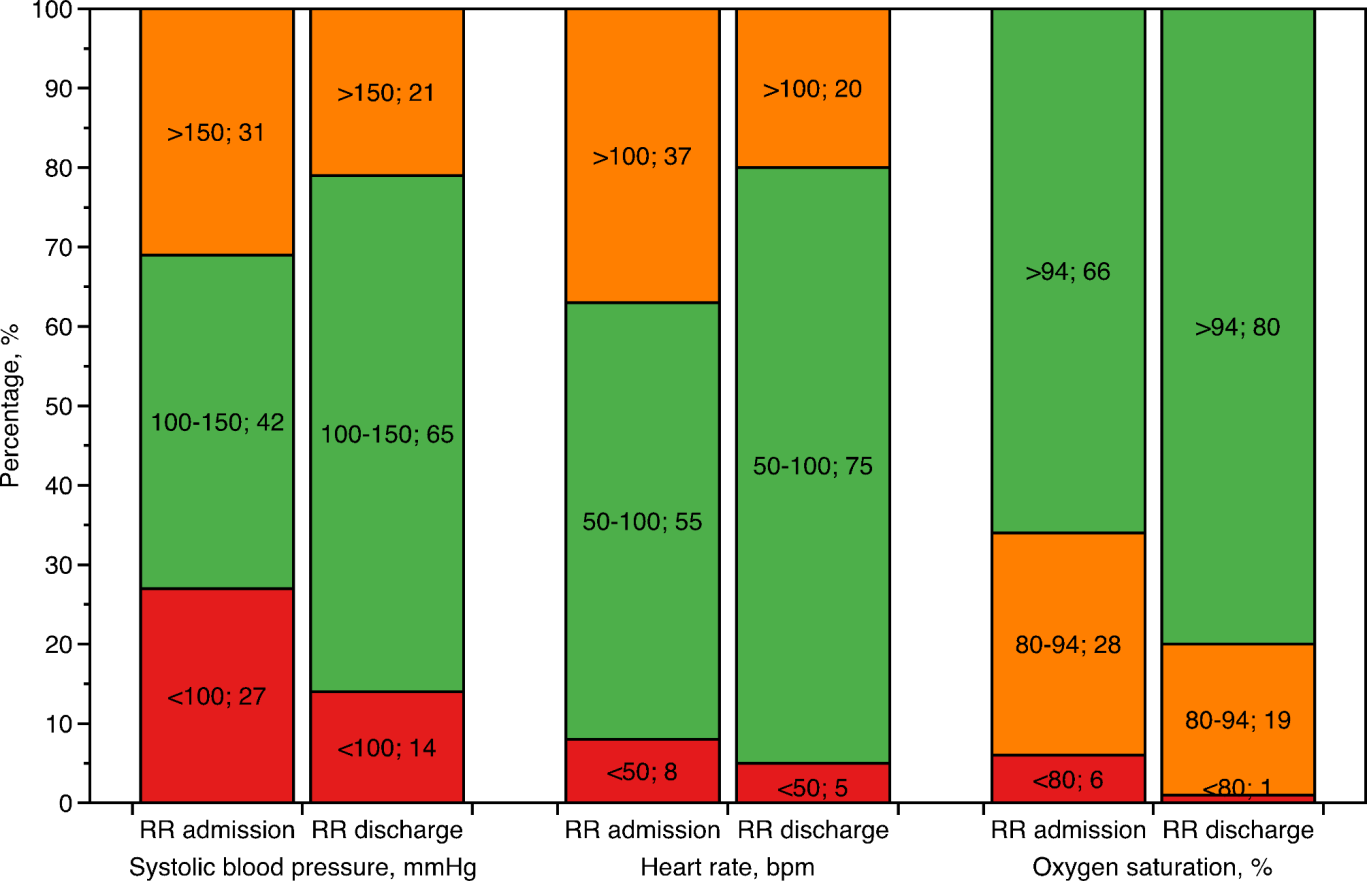
Vital signs at resuscitation room (RR) admission and discharge. Bar showing percentage (%) of patients within the described area of systolic blood pressure, heart rate, and oxygen saturation

Of the three vital signs blood pressure, heart rate, and peripheral oxygen saturation, 58, 45, and 34% of patients, respectively, showed vital functions outside the predefined target ranges despite out-of-hospital EMS care (Fig. 1). A total of 30.6% of all patients had a Glasgow Coma Scale (GCS) < 9 at hospital admission. Changes in vital signs from admission to the resuscitation room to discharge are presented in Fig. 1 and Table S2.

### Diagnoses

The main diagnoses verified at hospital discharge leading to ED admission are listed in Table 3. When categorized, most patients were treated for neurologic, cardiovascular, pneumological emergencies or others with 29.3%, 28.3%, 19.5%, and 22.9%, respectively.

**Table 3:** Emergency department diagnoses spectrum in studies concerning resuscitation room management of patients suffer from non-traumatic critical illness. Comparison between the current OBSERvE-DUS study and three studies from other German sides

Diagnoses [n, (%)]	OBSERvE-DUS n = 621	OBSERvE-1 [6] n = 532		OBSERvE 2 [7] n = 457	Kreß et al. [4] n = 193	p (χ²-test)
**Neurological emergencies**	**182 (29.3)**	**119 (22.4)**		**115 (25.2)**	**38 (19.7)**	^a^0.0079, ^b^0.1367, ^c^0.0088
stroke	109 (17.6)	33 (6.2)		21 (4.6)	6 (3.1)	^a^<0.0001, ^b^<0.0001, ^c^<0.0001
convulsive seizure	37 (6.0)	35 (6.6)		35 (7.7)	10 (5.2)	^a^0.6755, ^b^0.2708, ^c^0.6784
intracerebral haemorrhage	33 (5.3)	39 (7.3)		44 (9.6)	22 (11.4)	^a^0.1613, ^b^0.0067, ^c^0.0032
meningitis	2 (0.3)	0 (0.0)		0 (0.0)	0 (0.0)	^a^0.2063, ^b^0.2415, ^c^0.4465
idiopathic Parkinson’s syndrom	1 (0.2)	0 (0.0)		0 (0.0)	0 (0.0)	^a^0.3023, ^b^0.3390, ^c^0.5143
vigilance disorder	0 (0.0)	12 (2.3)		15 (3.3)	0 (0.0)	^a^0.0001, ^b^<0.0001, ^c^=1.000
**Pulmonary emergencies**	**121 (19.5)**	**115 (21.6)**		**111 (24.3)**	**54 (28.0)**	^a^0.3785, ^b^0.0583, ^c^0.0121
pneumonia	119 (19.2)	99 (18.6)		103 (22.5)	52 (26.9)	^a^0.7955, ^b^0.1857, ^c^0.0219
respiratory haemorrhage	1 (0.2)	2 (0.3)		0 (0.0)	0 (0.0)	^a^0.7323, ^b^0.3390, ^c^0.5343
pneumothorax	1 (0.2)	1 (0.2)		1 (0.2)	2 (1.0)	^a^1.0000, ^b^1.0000, ^c^0.1194
aspiration	0 (0.0)	9 (1.7)		5 (1.1)	0 (0.0)	^a^0.0011, ^b^0.0088, ^c^1.000
bolus event	0 (0.0)	4 (0.7)		2 (0.4)	0 (0.0)	^a^0.0369, ^b^0.1149, ^c^1.000
**Cardiocascular emergencies**	**176 (28.3)**	**161 (30.3)**		**138 (30.2**)	**59 (30.6)**	^a^0.4568, ^b^0.4976, ^c^0.5381
cardiovascular arrest, unclear	62 (10.0)	14 (2.6)		20 (4.4)	11 (5.7)	^a^<0.0001, ^b^0.0006, ^c^0.0682
heart failure	45 (7.2)	67 (12.6)		36 (7.9)	24 (12.4)	^a^0.0020, ^b^0.6664, ^c^0.0233
cardiac arrhythmia	43 (6.9)	13 (2.4)		19 (4.2)	8 (4.1)	^a^0.0004, ^b^0.0601, ^c^0.1603
acute myocardial infarction	20 (3.2)	49 (9.2)		46 (10.1)	4 (2.1)	^a^<0.0001, ^b^<0.0001, ^c^0.4297
pulmonary embolism	6 (1.0)	18 (3.4)		6 (1.3)	0 (0.0)	^a^0.0047, ^b^0.6449, ^c^0.1634
**Other emergencies**	**142 (22.9)**	**137 (25.6)**		**93 (20.4)**	**42 (21.8)**	^a^0.2858, ^b^0.3264, ^c^0.7499
intoxications	28 (4.5)	39 (7.3)		37 (8.1)	11 (0.51)	^a^0.0425, ^b^0.0142, ^c^0.0090
trauma	3 (0.5)	0 (0.0)		0 (0.0)	0 (0.0)	^a^0.1026, ^b^0.1303, ^c^0.3253
Abdominal aortic aneurysm, ruptured	2 (0.3)	4 (0.7)		2 (0.4)	0 (0.0)	^a^0.3297, ^b^0.7813, ^c^0.4465
aortic dissection	2 (0.3)	7 (1.3)		5 (1.1)	0 (0.0)	^a^0.0516, ^b^0.1035, ^c^0.4465
drowning	0 (0.0)	3 (0.6)		1 (0.2)	0 (0.0)	^a^0.0533, ^b^0.2651, ^c^1.000
hyperthermia	0 (0.0)	2 (0.4)		1 (0.2)	0 (0.0)	^a^0.1148, ^b^0.2651, ^c^1.000
hypothermia	0 (0.0)	1 (0.2)		1 (0.2)	0 (0.0)	^a^0.2651, ^b^0.2651, ^c^1.000
psychiatric disease	0 (0.0)	4 (0.7)		4 (0.9)	0 (0.0)	^a^0.0369, ^b^0.0179, ^c^1.000
smoke inhalation	0 (0.0)	2 (0.4)		2 (0.4)	0 (0.0)	^a^0.1148, ^b^0.1149, ^c^1.000.
gastrointestinal bleeding	15 (2.4)	19 (3.6)		12 (2.6)	12 (6.2)	^a^0.2304, ^b^0.8149, ^c^0.0099
acute abdomen	6 (1.0)	0 (0.0)		0 (0.0)	2 (1.0)	^a^0.0208, ^b^0.0321, ^c^1.0000
intraabdominal haemorrhage	0 (0.0)	0 (0.0)		1 (0.2)	0 (0.0)	^a^1.000, ^b^0.2651, ^c^1.000
acute kidney failure	8 (1.3)	7 (1.3)		7 (1.5)	0 (0.0)	^a^1.0000, ^b^0.7813, ^c^0.1116
dehydration	1 (0.2)	2 (0.4)		7 (1.5)	0 (0.0)	^a^0.5308, ^b^0.0146, ^c^0.5343
urosepsis	32 (5.1)	13 (2.4)		6 (1.3)	0 (0.0)	^a^0.0176, ^b^0.0008, ^c^0.0014
sepsis	7 (1.1)	15 (2.8)		7 (1.5)	17 (8.8)	^a^0.0344, ^b^0.5623, ^c^<0.0001
disorders, others	38 (6.1)	19 (3.5)		11 (2.4)	12 (6.2)	^a^0.0416, ^b^0.0039, ^c^0.9597

^a^comparison between OBSERvE-DUS and OBSEvE-1, ^b^comparison between OBSERvE-DUS and OBSEvE-2, ^c^comparison between OBSERvE-DUS and Kreß et al., n.a. = not available

Of 73 out-of-hospital cardiac arrest (OHCA) patients, 52 patients (71.2%) showed return of spontaneous circulation (ROSC) on admission and 21 patients (28.8%) were admitted under ongoing cardiopulmonary resuscitation (CPR). In twelve additional cases, in-hospital cardiac arrest (IHCA) occurred in the resuscitation room. Of note, the proportion of OHCA in the OBSERvE-DUS study with 11.8% was smaller than in the OBSERvE-1 and 2 study with 17.1 and 19.9%, respectively. The ROSC rate at hospital admission with 71.2% was in the OBSERvE-DUS study higher than in the OBSERvE-1 and 2 study with 60.4 and 47.3%, respectively.

### Relocation sites and outcome

Most patients (n = 469, 75.5%) were transferred to an intensive care unit (ICU), including 64 (10.3%) patients that had to be transferred to another hospital. Prior to ICU admission, 23 patients (3.7%) underwent interventions (e.g., angiography, surgery). A proportion of 13.5% (n = 84) of patients were admitted to normal ward and 5.0% (n = 31) were discharged home. During treatment in the resuscitation room, 37 patients (6.0%) died.

The mean LOS in resuscitation room during initial care and mean LOS in ED, including waiting time until transfer to ICU or another ward, was 120 ± 101 and 415 ± 479 min, respectively. Hospital and ICU LOS for patients with and without cardiac arrest did not differ significantly from each other (Table 4).

At day 30 the mortality was 18.5% in the whole study cohort (Table 4). The 30-day mortality in patients suffered from cardiac arrest was significantly higher in comparison to patients without cardiac arrest (54.1% vs. 12.9%, p = 0.0001).

**Table 4:** Length of stay and outcome parameters in studies concerning resuscitation room management of patients suffer from non-traumatic critical illness. Comparison between the current OBSERvE-DUS study and three studies from other German sides

Length of stay and outcome parameters	OBSERvE-DUS	OBSERvE-1 [6]		OBSERvE 2 [7]	Kreß et al. [4]	p (*Student-t-test, #χ²-test)
LOS Resuscitation room (min, MV ± SD)	120 ± 101	34 ± 24 ^a^		31 ± 22^b^	148 ± 203^c^	^a,*^<0.0001,^b,*^<0.0001, ^c,*^0.0105
LOS ED (min, MV ± SD)	415 ± 479	53 ± 34 ^a^		41 ± 24 ^b^	148 ± 203 ^c^	^a,*^<0.0001, ^b,*^<0.000,^c,*^<0.0001
LOS ICU (d, MV ± SD)	7 ± 10	6 ± 8 ^a^		8 ± 11 ^b^	not reported	^a,*^0.0640,^b,*^0.1203,^c^n.a.
LOS hospital (d, MV ± SD)	9 ± 12	11 ± 10 ^a^		12 ± 14 ^b^	not reported	^a,*^0.0024,^b,*^0.0002,^c^n.a.
30-day mortality, all [n, (%)]	115 (18.5)	181 (34.4) ^a^		166 (36.3) ^b^	56 (29.0) ^c^	^a,#^<0.0001,^b,#^<0.0001, ^c,#^0.0018
30-day mortality, CPR patients [n, (%)]	46 (54.1)	81 (72.7) ^a^		80 (79.2) ^b^	not reported	^a,#^<0.0001, ^b,#^<0.0001,^c,#^n.a.
30-day mortality no-CPR patients [n, (%)]	66 (12.9)	100 (24.0) ^a^		86 (24.2) ^b^	not reported	^a,#^<0.0001, ^b,#^<0.0001,^c,#^n.a.

LOS = length of stay; ED = emergency department; ICU = intensive care unit,

CPR = cardiopulmonary resuscitation, ^a^comparison between OBSERvE-DUS and OBSEvE-1, ^b^comparison between OBSERvE-DUS and OBSEvE-2, ^c^comparison between OBSERvE-DUS and Kreß et al.

## Discussion

In the present OBSERVE-DUS study, we retrospectively analysed the management of critically ill non-traumatic patients in the resuscitation room of a German tertiary university hospital. Structured resuscitation room care was required in 1.5% of all ED patients. Our study adds to the data on the epidemiology and management of critically ill non-traumatic patients in Germany. A medical need for structured care and training concepts for critically ill non-traumatic patients can be derived from the emerging picture and by international studies [10, 11].

Although study design as well as ED caseloads were different, our findings were in line with the three German studies (Table 1). The incidence of critically ill non-traumatic patients in the OBSERvE-DUS study with 1.5% was comparable with these previous investigations from German EDs with an incidence range between 1.0 and 1.6% [4, 6, 7]. In line with these investigations, the patients’ characteristics were comparable for gender, and age showed significant but minimal differences [4, 6, 7]. Taking these findings together, there is growing evidence that critically ill non-traumatic patients were older than patients suffering from severe trauma [12].

Comparing the results of the OBSERvE-DUS study with these of the OBSERvE-1 and 2 studies, we found a rough conformity for the distribution of ABCDE associated problems. Therefore, we can state that the individual complaint rates vary, but the grouped presentations are similar overall. Confirmation of these findings also in the OBSERvE-DUS study suggests that predominantly neurological problems with vigilance impairment (D), circulatory failure (C), and respiratory insufficiency (B) leads to life-threatening situations in critically ill non-traumatic patients. Although the incidence is very low at 0.5%, trauma sequelae has always be considered and excluded (e.g., fall sequelae in syncope).

In line with previous investigations, we found in the OBSERvE-DUS study the need for a high level of resuscitation room care at all times of the day, especially at the weekend and outside of the core working time [6]. In contrast to OBSERvE-1, we observed a difference of admission rate between the days of the week, interestingly, here we found a higher admission rate at the weekend. The demand of all-day special ED treatment is addressed by Kreß et al. [4] as well as other international studies [13]. Keeping these findings in mind, it can be deduced from the available data that there must be 24/7 readiness for the care of critically ill non-traumatic patients.

A scoring or grading system for the severity of non-traumatic condition in the resuscitation room is still missing. The NACA score was used as surrogate parameter as an established out-of-hospital score for illness and injury severity. A proportion of 86.8% of patients fell into the categories between 4 to 6, indicating that they had already had severe systemic illness by the time of sudden cardiac arrest. In the OBSERvE-1 study and OBSERvE-2 study, a NACA score between 4 to 6 was reported in a comparable amount of 92% and 85.0%, respectively [6, 7]. A study focusing on coma patients fits these assessments of the patient population [5]. The present results justify the demand for structured developed and validated alarming criteria for the non-traumatological resuscitation room, which are not available so far.

Consistent with previous studies, the majority of critically ill non-traumatic patients were admitted to the ED resuscitation room by EMS in up to 93.1% of cases [6, 7]. In-hospital medical emergency teams, interhospital transport and walking emergencies required resuscitation room care on a much smaller proportion. The contribution of triage for early recognition and structuring of care for critically ill non-traumatic patients in the ED is essential. Although we used defined admission criteria for the retrospective detection of critically ill non-traumatic patients, structured resuscitation protocols must be defined to avoid under-triage. Comparable results were shown recently by Kümpers et al. [14] in a validation study on the V2iSiOn rule for facilitate pragmatic identification using objective parameters.

In line with previous investigations, most patients in the OBSERvE-DUS study had neurological or cardiovascular emergencies [4, 6, 7]. In addition, according to the ABCDE approach, respiratory problems were subsequently leading. Other diseases, sepsis, abdominal causes, and metabolic disorders were significantly less common in all compared collectives [4, 6, 7]. The small differences in the collectives may be explained by differences in the assignment of different diseases. For example, pneumonia may be listed under sepsis on the one hand and pulmonary disorders on the other. This ambiguity in the evaluation should be eliminated by a standardized recording in further multicenter prospective studies. But already the need for a standardized training program for non-traumatological resuscitation room management can be deduced. Its contents can be well based on the previous knowledge of the probable diseases. Other international studies also see the requirement for standardized procedures in ED for several diseases [10, 11, 13, 15].

In accordance with the critically ill patient collective, a variety of emergency medical measures were applied in the ED resuscitation room. Leading among these measures were life-saving measures to eliminate airway and ventilation problems (e.g., non-invasive ventilation, airway management and invasive ventilation), and to stabilize the cardiovascular condition (e.g., arterial lines, central venous catheters, application of catecholamines). These findings were in line with the results from other studies investigating critically ill non-traumatic patients [4, 6, 7]. Also based on these findings, the training system already mentioned above must align itself accordingly.

Despite the EMS treatment, a high proportion of patients were still in extremis at ED resuscitation room admission using predefined target areas of blood pressure, heart rate and oxygen saturation as surrogate parameters. The special contribution of resuscitation room care was already demonstrated by optimizing blood pressure, heart rate, and oxygen saturation as surrogate parameters in previous studies [6]. In the OBSERvE-DUS study, we were also able to confirm the important contribution to successful emergency care for critically ill non-traumatic patients using the same method.

The outcome of patients in German ED is unclear. The 30-day mortality rate of patients never suffer from OHCA or IHCA during the resuscitation room course was 12.9% lower as in previous studies (24%) [4, 6, 7]. These findings were also consistent with these of several sepsis studies [16–19).

Moreover, the 30-day survival rate of patients with cardiac arrest in the OBSERvE-DUS study was higher with 45.9% than the results from single-center und registry-based studies [6, 7, 20].

The OBSERvE-1 and − 2 studies and the OBSERvE-DUS study used the same definition of OHCA according to the Utstein style, and were conducted in the same kind of EMS system with paramedics and physicians staffed EMS vehicles. However, in the OBSERvE-DUS study, resuscitation room management was performed in a certified cardiac arrest center, which was not developed and implemented in the OBSERvE-1 and − 2 study sites. The EuReCA ONE registry study included many EMS systems across Europe and also EMS systems with paramedics and paramedic/physician staffed ambulances.

An important difference was found comparing the results for LOS in the resuscitation room and LOS in ED in the presented OBSERvE-DUS study with other investigations [4, 6, 7]. These results show that the available ICU capacity at our facility is significantly more limited than at other sites. This means for our ED that intensive care measure has to be implemented on a much longer scale than at other sites. From this point of view, more pronounced intensive care expertise has to be applied to the care of the patients in our resuscitation room (e.g., differentiated ventilation patterns, adapted catecholamine therapy).

Taking into account all the above points, the patient population of critically ill non-traumatic patients represents high-risk patients in the resuscitation room of an ED and the following points should be developed in the future to optimize patient safety and the treatment outcome of these patients: [1] establishment of a registry for a multicenter registration of critically ill non-traumatic patients, if possible, [2] adaptation of the existing equipment of an ED resuscitation room to the requirements of the resuscitation room management of critically ill non-traumatic patients, [3] creation of a special care concept for the initial treatment and diagnostics for critically ill non-traumatic patients, and [4] gradual adaptation of the care concepts according to the findings of further studies.

Our investigations suffer from some limitations. At first, this is a retrospective single-center cohort study. With approximately 40,000 patient contacts, our ED is relatively large in a national comparison. However, our results may not be fully transferable to other locations. The data were extracted retrospectively and not collected simultaneously to the resuscitation room care. There is certainly a documentation bias regarding the interventions carried out, with a possible documentation deficit. Against this background, we assume that considerably more interventions were carried out. Our statistical processing of our extracted data, we deliberately chose to present processed parametric tests. The study results with which we want to compare our data were published only with the parametric data given. In order to create a comparability between the datasets, we have also opted for this.

## Conclusion

The data describe the care of critically ill non-traumatic patients in the resuscitation room. Critically ill non-traumatic patients suffer from a high mortality rate and require comprehensive diagnostic procedures in the resuscitation room. Life-saving interventions are frequently used. An impressive number of patients require structured and well-organized care in the ED resuscitation room. Based on these data, algorithms for the structured care of critically ill non-traumatic patients need to be developed.

## Electronic supplementary material

Below is the link to the electronic supplementary material.

Supplementary Material 1

## Data Availability

The datasets generated and/or analyzed during the current study are available from the corresponding author on reasonable request.

## References

[1] Sieber F, Kotulla R, Urban B, Groß S, Prückner S (2020). Entwicklung der Frequenz und des Spektrums von Rettungsdiensteinsätzen in Deutschland. Notfall + Rettungsmedizin.

[2] Bernhard M, Ramshorn-Zimmer A, Hartwig T, Mende L, Helm M, Pega J (2014). [Management of critically ill patients in the resuscitation room. Different than for trauma?]. Anaesthesist.

[3] Bernhard M, Gries A (2010). [Interaction of emergency medical services and the emergency department–challenges in the acute care of seriously ill or injured patients]. Anasthesiol Intensivmed Notfallmed Schmerzther.

[4] Kress JS, Ruppel M, Haake H, Vom Dahl J, Bergrath S (2022). Short-term outcome and characteristics of critical care for nontrauma patients in the emergency department. Anaesthesist.

[5] Schmidt WU, Ploner CJ, Lutz M, Mockel M, Lindner T, Braun M (2019). Causes of brain dysfunction in acute coma: a cohort study of 1027 patients in the emergency department. Scand J Trauma Resusc Emerg Med.

[6] Bernhard M, Doll S, Hartwig T, Ramshorn-Zimmer A, Yahiaoui-Doktor M, Weidhase L (2018). Resuscitation room management of critically ill nontraumatic patients in a german emergency department (OBSERvE-study). Eur J Emerg Med.

[7] Grahl C, Hartwig T, Weidhase L, Laudi S, Petros S, Gries A (2022). Early in-hospital course of critically ill nontrauma patients in a resuscitation room of a german emergency department (OBSERvE2 study). Anaesthesiologie.

[8] Wolff JK, Beyer AK, Wurm S, Nowossadeck S, Wiest M (2018). Regional Impact of Population Aging on changes in individual self-perceptions of aging: findings from the german ageing survey. Gerontologist.

[9] Perkins GD, Jacobs IG, Nadkarni VM, Berg RA, Bhanji F, Biarent D et al. Cardiac arrest and cardiopulmonary resuscitation outcome reports: update of the Utstein Resuscitation Registry Templates for Out-of-Hospital Cardiac Arrest: a statement for healthcare professionals from a task force of the International Liaison Committee on Resuscitation (American Heart Association, European Resuscitation Council, Australian and New Zealand Council on Resuscitation, Heart and Stroke Foundation of Canada, InterAmerican Heart Foundation, Resuscitation Council of Southern Africa, Resuscitation Council of Asia); and the American Heart Association Emergency Cardiovascular Care Committee and the Council on Cardiopulmonary, Critical Care, Perioperative and Resuscitation. Circulation. 2015;132(13):1286 – 300. 10.1161/CIR.0000000000000144 25391522

[10] Gunnerson KJ, Bassin BS, Havey RA, Haas NL, Sozener CB, Medlin RP (2019). Association of an Emergency Department-Based Intensive Care Unit with Survival and Inpatient Intensive Care Unit admissions. JAMA Netw Open.

[11] Herring AA, Ginde AA, Fahimi J, Alter HJ, Maselli JH, Espinola JA (2013). Increasing critical care admissions from U.S. emergency departments, 2001–2009. Crit Care Med.

[12] Gather A, Grützner PA, Münzberg M (2019). Polytrauma im Alter – Erkenntnisse aus dem TraumaRegister DGU®. Chirurg.

[13] Mohr NM, Wessman BT, Bassin B, Elie-Turenne MC, Ellender T, Emlet LL (2020). Boarding of critically ill patients in the Emergency Department. Crit Care Med.

[14] Rovas A, Paracikoglu E, Michael M, Gries A, Dziegielewski J, Pavenstadt H (2021). Identification and validation of objective triggers for initiation of resuscitation management of acutely ill non-trauma patients: the INITIATE IRON MAN study. Scand J Trauma Resusc Emerg Med.

[15] Haas NL, Whitmore SP, Cranford JA, Tsuchida RE, Nicholson A, Boyd C (2020). An Emergency Department-Based Intensive Care Unit is Associated with decreased hospital and intensive care unit utilization for Diabetic Ketoacidosis. J Emerg Med.

[16] Rivers ENB, Havstad S, Ressler J, Muzzin A, Knoblich B, Peterson E, Tomlanovich M. Early goal-directed therapy in the treatment of severe sepsis and septic shock. N Engl J Med 2001; 345 (19):1368-1377 10.1056/NEJMoa010307 11794169

[17] Investigators A, Group ACT, Peake SL, Delaney A, Bailey M, Bellomo R (2014). Goal-directed resuscitation for patients with early septic shock. N Engl J Med.

[18] Pro CI, Yealy DM, Kellum JA, Huang DT, Barnato AE, Weissfeld LA (2014). A randomized trial of protocol-based care for early septic shock. N Engl J Med.

[19] Mouncey PR, Osborn TM, Power GS, Harrison DA, Sadique MZ, Grieve RD (2015). Trial of early, goal-directed resuscitation for septic shock. N Engl J Med.

[20] Grasner JT, Lefering R, Koster RW, Masterson S, Bottiger BW, Herlitz J (2016). EuReCa ONE-27 nations, ONE Europe, ONE Registry: a prospective one month analysis of out-of-hospital cardiac arrest outcomes in 27 countries in Europe. Resuscitation.

