# Supplementary material for: Resuscitation room management of patients with non-traumatic critical illness in the emergency department (OBSERvE-DUS-study)
Source: BMC Emerg Med. 2023 Apr 17;23:43. doi: 10.1186/s12873-023-00812-y (PMC10111786; doi:10.1186/s12873-023-00812-y)
Supplement: Supplementary file 1 — Supplementary Material 1 [file 12873_2023_812_MOESM1_ESM.docx]

**Supplemental Material**

| **Table S1: Resuscitation room admission criteria for non-traumatic critically ill patients according to the ABCDE approach** | |
| --- | --- |
| **Problem**: | **Examples:** |
| **A: Airway** | enoral swelling (e.g., angioedema, haematoma of the tongue, allergic reaction, abscess), free airway at risk, out-of-hospital airway management (including all alternative devices) |
| **B: Breathing** | respiratory insufficiency with high respiratory rate (suggested/ /ventilation or rapid deterioration) and low oxygenation level using pulse oximetry (SpO2 ≤85% under ambient air, or ≤92% under oxygen supply), Patients requiring rapid airway management (announcement of difficult airway) patients under non-invasive and mechanical, breathing ventilation, Inhalation trauma (thermic/toxic/chemical) |
| **C: Circulation** | circulatory insufficiency (e.g., hypotension SBP <80 mmHg, heart rate ≤40 or ≥180 bpm shock of each origin), after and ongoing cardiopulmonary resuscitation, relevant cardiac arrhythmia (e.g., AV block III°, ventricular tachycardia, ventricular fibrillation), bleeding (e.g., esophageal varices) |
| **D: Disability** | relevant altered mental state (GCS ≤9), intracerebral bleeding/stroke with acute neurological, symptoms or/and other ABCDE problem, lysis indication |
| **E: Environment** | intoxication with ABCDE problem, other critical condition, hypothermia ≤32°C, hyperthermia ≥40°C |

| **Table S2: Vital functions at resuscitation room admission and discharge** | | | |
| --- | --- | --- | --- |
|  | **Resuscitation room admission**  (n=621) | **Resuscitation room discharge**  (n=592) | **P (*Student-t-test,  ^#^χ²-test)** |
| **Oxygen saturation** (%), MV±SD, Median (min-max) | 94±8, 96 (52-100) | 97±4, 98 (64-100) | *<0.0001 |
| **Heart rate** (bpm), MV±SD, Median (min-max) | 95±35, 90 (28-275) | 83±22, 80 (30-152) | *<0.0001 |
| **Systolic blood pressure** (mmHg), MV±SD, Median (min-max) | 129±45, 124 (16-264) | 128±27, 126 (37-208) | *0.660 |
| **Shock** (<90 mmHg) [n, (%)] | 127 (21.3) | 30 (5.9) | **^#^**<0.0001 |
| **Ongoing chest compression** [n (%)] | 21 (23.3) | 0 (0) | **^#^**<0.0001 |
| **Temperature** **tympana**l (°C), MV±SD, Median (min-max) | 36.2±1.5, 36 (24.3-40.8) | 35.3±0.6, 35 (34.8-35.9) | *0.299 |
| **Respiratory rate** (x/min), MV±SD, Median (min-max) | 22±13, 18 (5-36) | 23±12, 21 (3-39) | *0.206 |
| MV = mean value; SD = standard deviation | | | |
